# Menstrual, reproductive and hormonal factors and thyroid cancer: a hospital-based case–control study in China

**DOI:** 10.1186/s12905-020-01160-w

**Published:** 2021-01-06

**Authors:** Meng Wang, Wei-Wei Gong, Qing-Fang He, Ru-Ying Hu, Min Yu

**Affiliations:** Zhejiang Provincial Center for Disease Control and Prevention, 3399 Binsheng Road, Hangzhou, 310051 China

**Keywords:** Reproductive history, Pregnancy, Thyroid cancer, Case–control study

## Abstract

**Background:**

There have been considerable studies on the effects of reproductive factors on thyroid cancer risk, while findings are inconsistent. In this analysis, we aimed to investigate the associations between menstrual, reproductive and hormonal factors with thyroid cancer occurrence in a population of Chinese women.

**Methods:**

Using data from a 1:1 matched case–control study performed between 2015 and 2017 in Zhejiang Province of China, a second analysis of 2261 pairs of female subjects was conducted. The possible effects for thyroid cancer were evaluated in logistic regression models by odds ratios (ORs) with 95% confidence intervals (CIs).

**Results:**

Later age at first pregnancy (for > 25 vs. ≦ 20 years, OR: 0.47, 95% CI 0.23–0.96) and longer duration of breast feeding (for 6–12 vs. ≦ 6 months, OR: 0.49, 95% CI 0.24–0.98) were significantly associated with decreased occurrence of thyroid cancer, while no trend was observed. Stratified by age at enrollment, only the association with duration of breast feeding remained significant, but limited to younger women (≦ 50 years).

**Conclusions:**

Our results suggested that women with later age at first pregnancy or longer breast feeding duration were less likely to have thyroid cancer. These findings supported an influence role of reproductive factors in thyroid cancer risk.

## Background

Thyroid cancer is the most common cancer of the endocrine system [1] and the incidence has been increasing worldwide for the last decades [2]. In China, between 2000 and 2011, a dramatic rise in thyroid cancer incidence was observed among national female population, with an annual percentage change (APC) of 20.1% [3]. The rise in incidence may be attributed to the improved diagnostic techniques and over diagnosis in certain parts of the world [4, 5]. However, in 2017, the American College of Radiology (ACR) committees has published the Thyroid Imaging, Reporting and Data System (TI-RADS) guidelines to provide guidance regarding management of thyroid nodules on the basis of their ultrasound appearance [6]. Thus, a true increase in incidence also exists, which could be due to elevated exposure risks [7, 8].

Epidemiological research has identified ionizing radiation and benign thyroid disease as established risk factors for thyroid cancer [9]. Some other environmental and lifestyle factors, such as high body mass index (BMI), as well as low or high iodine intake and sleep disturbance were also reported [5, 10, 11]. However, in actual, the etiology of thyroid cancer remains largely unknown. It has long been suggested that the thyroid cancer incidence is much higher in women than in men [3, 12]. Furthermore, previous studies showed that the female-to-male incidence ratio is found to be the largest during the years between menarche and menopause [13], which support an influence role of menstrual, reproductive or hormonal factors in the etiology of thyroid cancer. To further confirm the hypothesis, considerable studies with different design have been conducted on this topic, while the observations were weak and inconsistent [14–16]. Besides, the above evidence was mainly from western world and other Asia counties, but limited studies were seen among Chinese female population. Between 2015 and 2017, we conducted a hospital-based case–control study in Zhejiang Province to explore the associated factors for thyroid cancer. In the study, self-reported data on menstrual, reproductive, and hormonal factors were collected, which provided us an opportunity to investigate the associations with thyroid cancer in Chinese women.

## Methods

### Study subjects

To explore the associations of diabetes mellitus and other factors with thyroid cancer, a 1:1 matched hospital-based case–control study was performed in 7 counties (Lucheng, Pingyang, Cangnan, Cixi, Nanhu, Changxing and Yongkang) of Zhejiang Province. Case subjects were eligible if they were first primary thyroid cancer diagnosed in hospitals from July 2015 to December 2017. All the cases were identified by physician review of medical records and pathology reports. Notably, among the cases, those who had a history of any cancer or were unable/unwilling to complete the study questionnaire were excluded. In the current study, thyroid cancer was further identified based on ICD-10 and referred to malignant neoplasm of thyroid gland (C73). In the same area and same year, control subjects were selected from thyroid-healthy examinees who underwent thyroid function tests and thyroid ultrasound screening in the annual routine physical examination, which was conducted in local hospitals. In Zhejiang Province, authorized by the government, fixed local hospitals will organize physical examination covering all the insured residents in community or village once a year. We excluded subjects with abnormal levels of free triiodothyronine, free thyroxine, and thyroid stimulating hormone (TSH), respectively. Besides, we excluded those whose ultrasound findings were suspicious for malignancy. Control subjects who had a history of any cancer or were unable/unwilling to complete the study questionnaire were also excluded. Finally, case subjects were matched to control subjects by age (plus or minus three years) and sex with 1:1.

### Estimation of the sample size

In our study, the cases and controls were selected by 1:1, so we calculated the sample size with the following special formula.

$$M = m/(p_{0} q_{1} + p_{1} q_{0} )$$

Among them,

$$m = {{\left[ {U_{\alpha } /2 + U_{\beta } \sqrt {p(1 - p)} } \right]^{2} } \mathord{\left/ {\vphantom {{\left[ {U_{\alpha } /2 + U_{\beta } \sqrt {p(1 - p)} } \right]^{2} } {\left( {p - \frac{1}{2}} \right)^{2} }}} \right. \kern-\nulldelimiterspace} {\left( {p - \frac{1}{2}} \right)^{2} }}$$

$$p = OR/(1 + OR) \approx RR/(1 + RR)$$

$$p_{1} = \frac{{p_{0} OR}}{{1 + p_{0} (OR - 1)}}$$

$$q_{1} = 1 - p_{1} ,\quad q_{0} = 1 - p_{0}$$

*P*_0_: The estimated diabetes exposure rate in control group; *P*_1_: The estimated diabetes exposure rate in case group; We presumed that: *P*_0_ = 0.07, OR = 2, *α* = 0.05, *β* = 0.10.

Consequently, *M* = 398 in each county, and actually, 2937 pairs of subjects were recruited in 7 counties.

### Questionnaire

With a designed questionnaire (Additional file 1), the interviewer collected all relevant information face-to-face at enrollment. The questionnaire was completed within two months after each pair of case and control was successfully matched. The questionnaire design went through literature review, panel discussion, check and approval by experts and revision after pilot study. Especially, to obtain the relatively high reliability of the questionnaire, we have reviewed literature and discussed in panel about the setting of questions. Besides, using the Delphi method, we invited relevant experts to check and revise the questionnaire. Finally, we also conducted several rounds of pilot studies to make sure the test–retest reliability was high. The same structured questionnaire was administered to each pair of subjects by the same trained interviewer. The questionnaire was used to collect the information on socio-demographic characteristics, individual history and family history of chronic diseases, lifestyle behaviors, environmental hazardous substances exposure, and dietary habits, etc. The present investigation focused on menstrual, reproductive and hormonal factors, including age at menarche, regularity of menstrual cycle, dysmenorrheal history, age at menopause, number of pregnancies, age at first/last pregnancy, outcome of the first pregnancy, breast feeding duration, use of oral contraceptives, hormone therapy, and history of hysterectomy and oophorectomy.

### Statistical analysis

Descriptive statistics were used to describe the baseline characteristics of female subjects with frequency and proportion. Univariate conditional logistic regression models were performed to examine the relationships of the covariates with the thyroid cancer.

To examine the associations between menstrual, reproductive and hormonal factors with thyroid cancer, we conducted four multivariable conditional logistic regression models to adjust for the covariates, including age (continuous), education level (no formal/primary school, middle/high school, college/university/postgraduate), average monthly household income (≦ 2000, 2000–5000, > 5000 Yuan), marriage status (unmarried, married), history of goiter (yes, no), history of nodules (yes, no), alcohol drinking (never, occasional, current regular), and BMI. Notably, BMI (kg/m^2^) was calculated as weight divided by the square of height. According to the Chinese adult BMI classification proposed by the Working Group on Obesity in China in 2001 [17], subjects were categorized as underweight (< 18.5), normal weight (18.5–23.9), overweight (24.0–27.9), and obesity (≥ 28.0). The possible effects were showed with odds ratios (ORs) and their 95% confidence intervals (CIs). In model 1, only age (continuous) was adjusted. In model 2, age (continuous) and other socio-demographic characteristics including education level, average monthly household income, marriage status, and history of goiter and nodules were included. Model 3 adjusted for model 2 plus the health behavior of alcohol intake. Model 4 adjusted for model 3 plus BMI. In model 4, the tests for trend were calculated using linear-by-linear association chi square test. Subgroup analyses were performed stratified by age (≦ 50, > 50 years) in multivariable logistic regression models and only the final results (model 4) were showed. All analyses were based on the two-sided 5% level of significance and performed using SAS statistical package (version 9.2, SAS Institute, Inc., Cary, NC, USA).

## Results

A total of 2937 pairs of subjects participated in the case–control study. Among them, there were 2261 pairs (77.0%) of females. In the present study, based on the study purposes, only the female subjects were available in the analysis process. Among the 2261 thyroid cancer cases, there were 2074 papillary cancers, 72 follicular cancers, 13 medullary cancers, 16 undifferentiated cancers and 86 other type. The mean age in female case and control subjects was 49.44 ± 1.16 years and 49.32 ± 1.18 years, respectively. Frequencies and proportions of baseline characteristics among case and control subjects were showed in Table 1. Compared to control subjects, case subjects were more likely to be overweight (*P* = 0.002), have goiter (*P* < 0.001) and nodules (*P* < 0.001) and less likely to be underweight (*P* = 0.012), have higher household income (> 5000 Yuan, *P* = 0.001), intake alcohol occasionally (*P* < 0.001) and current regularly (*P* = 0.013).

**Table 1:** The baseline characteristics of female subjects and their relationships with thyroid cancer

Factors	Cases (*N* = 2261; %)	Controls (*N* = 2261; %)	*P*	OR	95% CI
Age (Years)	49.44 ± 1.16	49.32 ± 1.18		Matched	
Education level					
No formal/primary school	985 (43.56)	968 (42.81)		Ref	
Middle/high school	913 (40.38)	904 (39.98)	0.658	0.96	0.82–1.13
College/university/postgraduate	359 (15.88)	384 (16.98)	0.136	0.82	0.64–1.06
Marriage status					
Unmarried	105 (4.64)	91 (4.02)		Ref	
Married	2151 (95.13)	2170 (95.98)	0.163	0.75	0.51–1.12
Average monthly household income (Yuan)					
≤ 2000	1646 (72.80)	1598 (70.68)		Ref	
2000–5000	271 (11.99)	242 (10.70)	0.947	1.01	0.81–1.25
> 5000	341 (15.08)	415 (18.35)	0.001	0.69	0.55–0.85
Body mass index (kg/m^2^)					
Normal weight (18.5–23.9)	1298 (57.41)	1416 (62.63)		Ref	
Underweight (< 18.5)	79 (3.49)	122 (5.40)	0.012	0.67	0.49–0.91
Overweight (24.0–27.9)	652 (28.84)	581 (25.70)	0.002	1.26	1.09–1.45
Obese (≥ 28.0)	157 (6.94)	142 (6.28)	0.073	1.25	0.98–1.59
Alcohol category					
Never	1848 (81.73)	1670 (73.86)		Ref	
Occasional	337 (14.90)	495 (21.89)	< 0.001	0.55	0.47–0.66
Current regular	76 (3.36)	96 (4.25)	0.013	0.66	0.48–0.92
History of goiter					
No	1346 (59.53)	633 (28.00)		Ref	
Yes	181 (8.01)	19 (0.84)	< 0.001	8.67	3.72–20.18
History of nodules					
No	1175 (51.97)	555 (24.55)		Ref	
Yes	352 (15.57)	96 (4.25)	< 0.001	2.35	1.66–3.32

*OR* odds ratio, *CI* confidence interval, *Ref.* reference

Percentages of each variable may not equal 100 because of missing data or rounding

Table 2 showed the results of multivariable models investigating the associations between menstrual, reproductive and hormonal factors with thyroid cancer. In model 1, longer breast feeding duration of 6–12 months and > 12 months were significantly associated with decreased occurrence of thyroid cancer, with the ORs were 0.69 (95% CI 0.55–0.88) and 0.68 (95% CI 0.52–0.90), respectively. Women with hormone therapy use (OR: 2.16, 95% CI 1.13–4.14) and hysterectomy (OR: 1.71, 95% CI 1.19–2.46) were more likely to have thyroid cancer. With further adjustment for other socio-demographic characteristics and health behavior of alcohol, both later age at menarche (≥ 17 vs. 13–14 years) and age at first pregnancy (> 25 vs. ≦ 20 years) were significantly associated with decreased occurrence of thyroid cancer in model 2 and 3. After adjustment for the BMI in model 4, women with later age at first pregnancy (> 25 vs. ≦ 20 years) and relatively longer duration of breast feeding (6–12 vs. ≦ 6 months) were less likely to have thyroid cancer, with the ORs were 0.47 (95% CI 0.23–0.96) and 0.49 (95% CI 0.24–0.98), respectively. In the final results, no trend was observed (all *P*-values > 0.05). Stratified analyses showed that the associations between menstrual, reproductive and hormonal factors with thyroid cancer were modified by age, and no interactions were found (all *P*-values > 0.05). The specific results were showed in Additional file 2: Table S1.

**Table 2:** ORs of thyroid cancer associated with menstrual, reproductive and hormonal factors

Factors	Cases (N = 2261)	Controls (N = 2261)	Model 1		Model 2		Model 3		Model 4	
			OR^†^	95% CI	OR^†^	95% CI	OR^†^	95% CI	OR^†^	95% CI
*Menstrual factors*										
Age at menarche (years)										
≦ 12	104	99	0.51	0.23–1.12	0.72	0.18–2.92	0.77	0.19–3.17	0.67	0.15–3.10
13–14	974	995	Ref		Ref		Ref		Ref	
15–16	857	859	0.90	0.69–1.18	0.73	0.46–1.17	0.72	0.45–1.16	0.79	0.48–1.30
≥ 17	304	290	0.85	0.61–1.19	**0.54**	**0.29–0.99**	**0.52**	**0.28–0.97**	0.59	0.31–1.12
* P* for trend									0.650	
Had regular menstrual cycles										
No	187	182	1.27	0.82–1.96	0.66	0.32–1.40	0.63	0.30–1.34	0.61	0.28–1.35
Yes	2054	2067	Ref		Ref		Ref		Ref	
Had dysmenorrhea										
No	1757	1719	Ref		Ref		Ref		Ref	
Yes	495	535	0.92	0.70–1.21	0.82	0.51–1.32	0.81	0.50–1.31	0.70	0.42–1.18
Age at menopause (years)										
≦ 44	68	64	0.97	0.60–1.58	1.08	0.49–2.39	1.03	0.46–2.30	0.98	0.42–2.29
45–49	249	224	1.03	0.77–1.39	0.98	0.57–1.67	0.96	0.56–1.65	0.92	0.52–1.61
50–51	307	315	Ref		Ref		Ref		Ref	
≥ 52	307	303	1.07	0.82–1.39	0.90	0.57–1.41	0.86	0.54–1.36	0.89	0.55–1.44
* P* for trend									0.496	
*Reproductive factors*										
Number of pregnancy										
1	613	631	Ref		Ref		Ref		Ref	
2	771	796	0.96	0.77–1.18	0.63	0.36–1.12	0.62	0.35–1.11	0.67	0.37–1.23
≥ 3	774	728	1.04	0.81–1.34	0.64	0.34–1.20	0.62	0.33–1.18	0.66	0.34–1.28
* P* for trend									0.222	
Age at first pregnancy (years)										
≦ 20	336	325	Ref		Ref		Ref		Ref	
20–25	1387	1354	0.99	0.78–1.25	1.03	0.63–1.69	1.03	0.63–1.70	0.96	0.57–1.61
> 25	426	465	0.87	0.64–1.19	**0.47**	**0.24–0.93**	**0.47**	**0.23–0.94**	**0.47**	**0.23–0.96**
* P* for trend									0.200	
Outcome of the first pregnancy										
Live birth	1920	1946	Ref		Ref		Ref		Ref	
Miscarriage	81	80	1.13	0.78–1.64	0.93	0.37–2.33	0.94	0.37–2.35	0.85	0.33–2.20
Abortion	125	95	1.39	0.99–1.96	0.81	0.31–2.07	0.78	0.30–2.03	0.80	0.30–2.09
Stillbirth or ectopic	22	18	1.28	0.65–2.53	1.36	0.34–5.53	1.37	0.34–5.57	1.37	0.33–5.62
Age at last pregnancy (years)										
≦ 25	806	777	Ref		Ref		Ref		Ref	
25–30	883	924	0.97	0.81–1.17	1.17	0.76–1.81	1.17	0.75–1.81	1.21	0.77–1.90
> 30	418	409	1.04	0.81–1.33	1.54	0.86–2.76	1.50	0.83–2.73	1.63	0.88–3.00
* P* for trend									0.667	
Duration of breast feeding (months)										
≦ 6	293	234	Ref		Ref		Ref		Ref	
6–12	1040	1087	**0.69**	**0.55–0.88**	0.52	0.26–1.03	0.53	0.27–1.05	**0.49**	**0.24–0.98**
> 12	674	685	**0.68**	**0.52–0.90**	0.67	0.31–1.44	0.68	0.31–1.46	0.60	0.27–1.31
* P* for trend									0.090	
*Hormonal factors*										
Oral contraceptive use										
No	1637	1667	Ref		Ref		Ref		Ref	
Yes	62	56	0.96	0.63–1.46	1.24	0.51–3.03	1.17	0.48–2.87	1.18	0.47–2.96
Hormone therapy use										
No	2204	2233	Ref		Ref		Ref		Ref	
Yes	57	28	**2.16**	**1.13–4.14**	2.24	0.53–9.56	2.06	0.47–9.06	1.47	0.30–7.38
Hysterectomy and oophorectomy status										
None	2107	2162	Ref		Ref		Ref		Ref	
Hysterectomy alone	119	74	**1.71**	**1.19–2.46**	1.36	0.74–2.52	1.37	0.74–2.54	1.45	0.77–2.73
Oophorectomy alone	28	15	2.01	0.93–4.34	1.84	0.42–7.96	1.74	0.41–7.41	1.93	0.43–8.72
Hysterectomy and oophorectomy	7	10	1.19	0.36–3.97	4.60	0.51–41.45	4.67	0.52–41.98	3.85	0.43–34.59

Trend test was calculated using the linear-by-linear association chi square test

*OR* odds ratio, *CI* confidence interval, *Ref.* reference

Bold numbers represent significant results

^†^Model 1 only adjusted for age (continuous); Model 2 adjusted for model 1 plus socio-demographic characteristics including education level, average monthly household income, marriage status, and history of goiter and nodules; Model 3 adjusted for model 2 plus the health behavior of alcohol intake; Model 4 adjusted for model 3 plus body mass index

## Discussion

In this case–control study, we investigated the associations between numerous menstrual, reproductive and hormonal factors with thyroid cancer occurrence in Chinese female population. According to the analyses, our results suggested decreased occurrence of thyroid cancer among women who reported a later age at first pregnancy or longer duration of breast feeding, although no trend was observed. In the stratified analysis by age at enrollment, differences in this pattern were noted, where the effects of age at first pregnancy diminished to be null and only the association with duration of breast feeding remained significant in younger women.

In the present study, later age at first pregnancy was significantly associated with lower occurrence of thyroid cancer in total women (for > 25 vs. ≦ 20 years, OR: 0.47, 95% CI 0.23–0.96), whereas the association was not significant in age subgroups (≦ 50 and > 50 years). In contrast, a population-based case–control study of young women below age 35 years performed in France reported a lower risk of thyroid cancer among those who had a first pregnancy after the age of 25 years (OR: 0.5, 95% CI 0.3–0.9) [18]. Several other studies on this topic have also been conducted, giving mixed results. A meta-analysis of prospective studies showed a directly opposite result that the increasing age at first pregnancy/birth was positively associated with thyroid cancer risk (SRR: 1.56, 95% CI 1.01–2.42) [19]. Parallel to this, findings from a population-based case–control study conducted in San Francisco Bay Area suggested an increase in risk with age at first full term pregnancy between 25 and 29 years (OR: 3.3, 95% CI 1.5–7.4) and ≥ 30 years (OR: 2.8, 95% CI 1.1–7.0), with reference to < 20 years, but these associations were restricted to younger women (< 45 years) [13]. Meanwhile, in some other studies have investigated this relationship of age at first pregnancy with thyroid cancer, non-significant higher or lower risks were also observed [20, 21]. As in the age at first pregnancy, the relationships between duration of breast feeding and thyroid cancer risk seemed to be inconclusive as well. In this study, we found decreased occurrence of thyroid cancer among women with relatively longer duration of breast feeding (for 6–12 vs. ≦ 6 months, OR: 0.49, 95% CI 0.24–0.98). Consistently, pooled result from a recent meta-analysis of cohort studies suggested that longer duration of breast feeding was associated with moderately decreased risk of thyroid cancer (RR: 0.7, 95% CI 0.51–0.95) [22]. Similar results concerning the protective effect of breast feeding was also confirmed in a case–control study conducted in France (OR per month: 0.7, 95% CI 0.5–1.0) [18]. However, a few other studies found little support for the association of breast feeding duration with risk of developing thyroid cancer [22–25]. Besides, it is noteworthy that, in a population-based case–control study conducted in western Washington State, the authors brought the recency of breast feeding into analysis and found that the risk was increased among parous women with breast feeding during the previous 5 years (for ≥ 12 vs. 0–1 months, RR: 2.9, 95% CI 1.5–5.5) [26]. In theory, the major differences in the associations of age at first pregnancy and breast feeding with thyroid cancer among published studies with respect to sample size, study design, population characteristics, definition and categorization of the exposures of interest. Based on previous studies, the potential mechanism behind the associations of later age at first pregnancy and longer breastfeeding duration with lower thyroid cancer risk might be the decreased level of estrogens, which could have a function in the proliferation of malignant thyroid cells [18, 27, 28]. In the current study, the absence of significant results across the majority of menstrual, reproductive and hormonal factors provided little support for their associations with thyroid cancer. Nevertheless, the lack of appreciable relationships with age at menarche and menopause, miscarriage and abortion or oral contraceptive and hormonal therapy use were also of importance, since they have been extensively investigated in previous studies [29–31].

Strengths of our study included relatively large number of women, all diagnosed incident thyroid cancer cases identified by physician in hospital and the availability of a range of menstrual, reproductive and hormonal factors.

However, some limitations should be considered. Firstly, the detailed information on exposures of interest was reported by women themselves and the recall bias inherent to case–control studies may be inevitable. Secondly, the lack of some information on the exposures, such as the menopausal status, recency of breast feeding, the type and duration of oral contraceptive and hormone therapy use, limited our ability to investigate their associations with thyroid cancer risks in depth. Thirdly, despite adjustment for a certain set of confounders, other potential risk factors for thyroid cancer may have affected the final results.

## Conclusions

In conclusion, our results suggested that later age at first pregnancy and longer breast feeding duration were significantly associated with the decreased occurrence of thyroid cancer, which might shed light on the etiology, monitoring and prevention of thyroid cancer among Chinese women. However, further evidence from prospective studies on the possible influence role of menstrual, as well as reproductive and hormonal factors in the risk of thyroid cancer among Chinese female population is warranted.

## Supplementary information

**Additional file 1**. The questionnaire used for this study.

**Additional file 2: Table S1**. ORs of thyroid cancer associated with menstrual, reproductive and hormonal factors, stratified by age at enrollment.

## Data Availability

The data can be accessed from the corresponding author upon justified request.

## Abbreviations

APC: Annual percentage change
BMI: Body mass index
TSH: Thyroid stimulating hormone
OR: Odds ratio
CI: Confidence interval
