# Supplementary material for: Menstrual, reproductive and hormonal factors and thyroid cancer: a hospital-based case–control study in China
Source: BMC Womens Health. 2021 Jan 6;21:13. doi: 10.1186/s12905-020-01160-w (PMC7789638; doi:10.1186/s12905-020-01160-w)
Supplement: Supplementary file 1 — Additional file 1. The questionnaire used for this study. [file 12905_2020_1160_MOESM1_ESM.docx]

**疾病危险因素监测调查表**

对子号 病例T/对照C 地区 序号

**个人编码**

被调查者姓名：

常住地址： 县/区 街道/乡镇 居委会/村

在本县（区）居住年限： 年

户籍地址： 县/区

联系电话：

调查日期 年 月 日 调查员签名：

核查日期 年 月 日 核查员签名：

**开场白**

您好！我们正在做的是有关于疾病危险因素的调查，主要了解您的健康状况、生活居住环境以及一些生活行为习惯。通过此调查，我们可以获得有关影响人体健康的危险因素，即存在于您身边的“隐形杀手”，为进一步做好疾病的预防和控制工作提供可靠的依据。调查不会占用您过多的时间，而且调查的内容都是保密的，我们承诺，您的个人信息不会公开。您的回答对我们的调查十分重要，希望您能支持我们的工作，真实准确的回答问题，配合我们顺利完成调查，这将是对我省公共卫生事业的莫大贡献。

谢谢！

**知情同意书**

本人已了解这次研究的目的，并对调查员的介绍感到满意，我自愿（或代表有关亲属）参加这项研究，并同意提供相关标本。

调查对象（或家属）签名：_____________

日 期: _____________

**疾病危险因素监测调查表**

调查开始时间（24小时制）： □□时□□分

| **A. 一般情况** | | | | **回答** |
| --- | --- | --- | --- | --- |
| A1 | **组别：** ⑴ 病例组 ⑵ 对照组 | | |  |
| A2 | **性别：** ⑴ 男 ⑵ 女 | | |  |
| A3 | **身份证号：**  □□□□□□□□□□□□□□□□□□ | | |  |
| A4 | **民族：** ⑴汉族 ⑵畲族 ⑶其它 | | |  |
| A5 | **文化程度：**  ⑴不识字或识字少 ⑵小学 ⑶初中 ⑷高中/技校/中专  ⑸大专 ⑹大学及以上 | | |  |
| A6 | **婚姻状况：** ⑴未婚 ⑵初婚 ⑶再婚 ⑷离婚 ⑸丧偶 ⑹其它 | | |  |
| A7 | **职业：**  ⑴农林牧渔水利业劳动者 ⑵生产、运输设备操作人员及有关人员  ⑶商业、服务业人员 ⑷国家机关、党群组织、企业、事业单位负责人  ⑸办事人员和有关人员 ⑹专业技术人员（医生、教师、科技人员）  ⑺军人 ⑻在校学生 ⑼学龄前儿童 ⑽未就业 ⑾家务  ⑿离退休人员**（请回答A7.1）**  ⒀其他 | | |  |
| **A7.1** | **你离退休之前的最后一份工作，主要从事哪方面的职业？**  ⑴农林牧渔水利业劳动者 ⑵生产、运输设备操作人员及有关人员  ⑶商业、服务业人员 ⑷国家机关、党群组织、企业、事业单位负责人  ⑸办事人员和有关人员 ⑹专业技术人员（医生、教师、科技人员）  ⑺军人 ⑻其他 | | |  |
| A7.2 | **是否曾从事以下工种？** ⑴是 ⑵否**（跳至A8）** | | |  |
| A7.3 | **具体工种：**⑴冶金 ⑵矿山 ⑶化工 ⑷建筑 ⑸机电 ⑹塑料 ⑺印染 ⑻纺织 ⑼制革 ⑽油漆 ⑾司机 ⑿厨师 | | |  |
| **A8** | **目前您的家庭共同生活的人口数** | | | □□人 |
| A9 | **目前您家庭经济平均月总收入（不回答填写9）** | | | 元 |
| A10 | **十年前您的家庭共同生活的人口数** | | | □□人 |
| A11 | **十年前您家庭经济平均月总收入（不回答填写9）** | | | 元 |
| A12 | **您目前是否参加医疗保险？** ⑴是 ⑵否**（跳至B1）** | | |  |
| A12.1 | **如果是，具体是哪些？** ⑴是 ⑵否  ⑴城镇职工基本医疗保险 □  ⑵城镇居民医疗保险 □  ⑶新型农村合作医疗 □  ⑷公费医疗 □  ⑸商业医疗保险 □ | | |  |
| **B. 疾病及家族史** | | | |  |
| B1 | **医生曾经诊断您患过下述疾病吗？** ⑴是 ⑵否 ⑼不详  **是否患病 确诊时间**   1. 单纯性甲状腺肿 □ □□□□年□□月□□日 2. 结节性甲状腺肿 □ □□□□年□□月□□日 3. 甲亢 □ □□□□年□□月□□日 4. 甲减 □ □□□□年□□月□□日 5. 甲状腺炎 □ □□□□年□□月□□日 6. 甲状腺良性肿瘤 □ □□□□年□□月□□日 7. 甲状腺结节 □ □□□□年□□月□□日 8. 高血压 □ □□□□年□□月□□日 9. 糖尿病 □ □□□□年□□月□□日 10. 血脂异常 □ □□□□年□□月□□日 11. 冠心病 □ □□□□年□□月□□日 12. 脑卒中 □ □□□□年□□月□□日 13. 恶性肿瘤，部位 □ □□□□年□□月□□日 14. 其它 □ □□□□年□□月□□日 | | |  |
| B2 | **主要慢性病家族史（可多选，没有填99）：**  亲属关系代码   1. 单纯性甲状腺肿 □□/□□/□□ 2. 结节性甲状腺肿 □□/□□/□□ 3. 甲亢 □□/□□/□□ 4. 甲减 □□/□□/□□ 5. 甲状腺炎 □□/□□/□□ 6. 甲状腺结节 □□/□□/□□ 7. 甲状腺癌 □□/□□/□□ 8. 肝癌 □□/□□/□□ 9. 胃癌 □□/□□/□□ 10. 大肠癌 □□/□□/□□ 11. 乳腺癌 □□/□□/□□ 12. 宫颈癌 □□/□□/□□ 13. 肺癌 □□/□□/□□ 14. 其它癌症 □□/□□/□□ 15. 糖尿病 □□/□□/□□   亲属关系代码：  01— 母亲 02—父亲 03—姐妹 04—兄弟 05—女儿 06—儿子  07- 祖父母 08- 外祖父母 09- 配偶 10- 叔姑 11- 舅姨 12- 其他 | | |  |
| **C. 吸烟情况** | | | |  |
| C1 | | **您现在是否吸烟？**  ⑴从不吸**（跳至C3）** ⑵已戒烟 ⑶现在吸，但不是每天吸 ⑷每天吸烟 | |  |
| C1.1 | | **您开始吸烟的年龄？** | | □□周岁 |
| C1.2 | | **开始每天吸烟的年龄？** | | □□周岁 |
| C1.3 | | **您过去或现在吸烟，扣除戒烟年数，共吸了几年？** | | □□年 |
| C1.4 | | **您过去或现在吸烟，平均每天吸多少支？** | | □□支 |
| C1.5 | | **您通常吸哪种烟？**  ⑴有过滤嘴 ⑵无过滤嘴 ⑶水烟 ⑷旱烟 ⑸混合 ⑹雪茄 | |  |
| C1.6 | | **您早晨醒来后多长时间吸第一支烟？**  ⑴>60分钟   ⑵31-60分钟    ⑶6-30分钟    ⑷≤ 5分钟 | |  |
| C1.7 | | **您是否在许多禁烟场所很难控制吸烟？** ⑴是 ⑵否 | |  |
| C1.8 | | **您认为哪一支烟您最不愿意放弃？** ⑴其他时间   ⑵早晨第一支 | |  |
| C1.9 | | **您早晨醒来后第1个小时是否比其他时间吸烟多？** ⑴是 ⑵否 | |  |
| C1.10 | | **您卧病在床时仍旧吸烟吗？** ⑴是 ⑵否 (3)不适用 | |  |
| C2 | | **您是否曾戒烟****（停止吸烟三个月以上）？** ⑴是 ⑵否**（跳至C3）** | |  |
| C2.1 | | **您戒烟，戒过几次烟？** | | □□次 |
| C2.2 | | **共戒烟多长时间？** | | □□年  □□月 |
| C2.3 | | **戒烟的主要原因是什么？** ⑴疾病 ⑵旁人劝阻 ⑶经济 ⑷认为烟有害 ⑸其它 | |  |
| **C3** | | **与您同住的家里人有几人吸烟？** **(无人吸烟填“00”，并跳转C3.4)**  | | □□人 |
| C3.1 | | **他们平均每人每天吸多少支？** | | □□支 |
| C3.2 | | **他们在家吸？** ⑴在家不吸 ⑵屋内有烟雾 ⑶满屋烟雾 ⑷在家吸不同室  | |  |
| C3.3 | | **您在有烟雾的环境中，与他们生活了多少年?**  | | □□年 |
| **C3.4** | | **在您工作场所办公室内有几人吸烟？** **(无人吸烟填“00”，并跳转D1)**   | | □□人 |
| C3.5 | | **他们平均每人每天吸多少支？**   | | □□支 |
| C3.6 | | **他们在办公室吸吗？** ⑴在办公室不吸 ⑵室内有烟雾 ⑶满屋烟雾  | |  |
| C3.7 | | **您在有烟雾的办公室内，与他们工作了多少年？** | | □□年 |
| **D. 饮酒/饮茶情况** | | | |  |
| **D1** | | **过去一年里，您大概多长时间喝一次酒？**  ⑴从不或几乎不饮酒  ⑵只在特殊场合下（如喜庆或节假日）偶尔喝  ⑶一年当中只在特定几个月里饮酒（如农忙或夏季），而其他季节一般不喝酒  ⑷一年当中不分季节，每个月都喝，但频度不到每周一次  ⑸一年当中不分季节，基本上每周至少喝一次酒**（跳至D2）** | |  |
| D1.1 | | **您以前是否曾经有过每周都喝酒，并且至少持续一年时间的经历？**  ⑴是 ⑵否**（跳至D2.3）** | |  |
| D1.2 | | **若是，停止饮酒大概在多久以前？** | | □□年  □□月 |
| D1.3 | | **停止饮酒的主要原因是什么？（跳至D2.3）** ⑴疾病 ⑵旁人劝阻 ⑶经济 ⑷其它 | |  |
| D2 | | **过去一年里，您平均每周大约有几天喝酒？** | | □天 |
| D2.1 | | **您大约从几岁开始养成每周都喝酒的习惯？** | | □□周岁 |
| D2.2 | | **您最常饮的酒种类及每日饮酒量（可同时选择几种酒类，没有饮用填00.0）**   \| 冬春季节 \| \| 夏秋季节 \| \| \| --- \| --- \| --- \| --- \| \| 种类 \| 数量 \| 种类 \| 数量 \| \| 白酒（≥40度） \| □□**.**□两 \| 白酒（≥40度） \| □□**.**□两 \| \| 白酒（<40度） \| □□**.**□两 \| 白酒（<40度） \| □□**.**□两 \| \| 啤酒(550ml，4度) \| □□**.**□瓶 \| 啤酒(550ml，4度) \| □□**.**□瓶 \| \| 黄酒、糯米酒 \| □□**.**□两 \| 黄酒、糯米酒 \| □□**.**□两 \| \| 葡萄酒 \| □□**.**□两 \| 葡萄酒 \| □□**.**□两 \| \| 其他( ) \| □□**.**□两 \| 其他( ) \| □□**.**□两 \| | |  |
| **D2.3** | | **过去一年中，您一次喝酒超过 3 两高度白酒，或 4 两低度白酒，或 3 瓶**  **半啤酒，或 6 个易拉罐啤酒，或 9 两黄酒/米酒，或 1 斤 8 两葡萄酒，或**  **3 斤半青稞酒的次数？** | | □□次 |
| D3 | | **您现在是否饮茶（茶饮料除外）？**  ⑴不或几乎不饮茶**（跳至D4）**  ⑵过去饮，但现在不饮**（跳至D4）**  ⑶偶尔饮茶**（即每月最多1—3次，每周少于一次）（跳至D4）**  ⑷经常饮茶**（每周最少饮一杯，持续6个月以上）** | |  |
| D3.1 | | **您习惯性每天饮茶多长时间了？** | | □□年 |
| D3.2 | | **请问您最喜欢饮哪种茶？**  ⑴绿茶类/茉莉花茶／龙井／白茶 ⑵乌龙茶类（铁观音、水仙等）  ⑶红茶类（包括砖茶，普洱） ⑷其他 | |  |
| D3.3 | | **请问您现在的饮茶量和频率？** | | □□杯/天，□天/周 |
| D3.4 | | **在过去一年里，您本人平均每月饮用的茶叶总量大约是多少？** | | □□两/月 |
| D3.5 | | **您喜欢喝浓茶还是淡茶?**  ⑴浓茶 ⑵适中 ⑶淡茶 | |  |
| D3.6 | | **您通常是喜欢喝滚烫的茶、热茶还是凉茶？** ⑴温茶/凉茶 ⑵热茶 ⑶烫茶 | |  |
| **D4** | | **在过去一年里，您大概多长时间喝一次咖啡？**  ⑴从不或几乎从不喝咖啡  ⑵只在特殊场合下（如节假日或做客时）偶尔喝  ⑶每月都喝，但频度不到每周一次  ⑷基本上每周都喝 | |  |
| **E. 身体活动**  下列问题是通常一周您进行各类身体活动（包括干农活、工作、家务、交通相关的身体活动、锻炼或娱乐活动等）的情况。请回答： | | | |  |
| E1 | | **工作、农业及家务性身体活动**  **在您的工作、农活及家务活动中，有没有高强度活动，并且活动时间持续10分钟以上？** （高强度活动是指如搬运重物、挖掘等需要付出较大体力，或引起呼吸、心跳显著增加的活动）  ⑴有 ⑵无**（跳至E1.5）** | |  |
| E1.1 | | **在您的工作、农活及家务活动中，通常一周内有多少天会进行上述高强度活动？** | | □天 |
| E1.2 | | **在您的工作、农活及家务活动中，通常一天内累计有多长时间进行上述高强度活动？**（每次活动时间若少于10分钟，则不计算在内） | | □□小时  □□分钟 |
| E1.3 | | **其中进行高强度家务活动天数为？（如填“0”，跳至E1.5)** | | □天 |
| E1.4 | | **在您的家务活动中，通常一天内累计有多长时间进行上述高强度活动？**（每次活动时间若少于10分钟，则不计算在内） | | □□小时  □□分钟 |
| **E1.5** | | **在您的工作、农活及家务活动中，有没有中等强度活动，并且活动时间持续10分钟以上？** （中等强度活动是指如锯木头、洗衣服、打扫卫生等需要付出中等体力，或引起呼吸、心跳轻度增加的活动）  ⑴有 ⑵无**（跳至E2）** | |  |
| E1.6 | | **在您的工作、农活及家务活动中，通常一周内有多少天会进行上述中等强度活动？** | | □天 |
| E1.7 | | **在您的工作、农活及家务活动中，通常一天内累计有多长时间进行上述中等强度活动？**（每次活动时间若少于10分钟，则不计算在内） | | □□小时  □□分钟 |
| E1.8 | | **其中进行中等强度家务活动天数为？（如填“0”，跳至E2)** | | □天 |
| E1.9 | | **在您的家务活动中，通常一天内累计有多长时间进行上述中等强度活动？**（每次活动时间若少于10分钟，则不计算在内） | | □□小时  □□分钟 |
| **E2** | | **交通性身体活动**（不包括上述活动）  **在您外出时，有没有步行或骑自行车持续至少10分钟的情况？**  ⑴有 ⑵无**（跳至E3）** | |  |
| E2.1 | | **通常一周内，您有多少天外出时步行或骑自行车持续至少10 分钟？** | | □天 |
| E2.2 | | **通常一天内，您步行或骑自行车多长时间？** | | □□小时  □□分钟 |
| **E3** | | **娱乐活动和锻炼**（不包括上述活动）  **您是否进行持续至少10 分钟，引起呼吸、心跳显著增加的高强度锻炼或娱乐活动吗？如长跑、游泳、踢足球等。**  ⑴有 ⑵无**（跳至E3.3）** | |  |
| E3.1 | | **通常一周内，您有多少天进行上述高强度的锻炼或娱乐活动？** | | □天 |
| E3.2 | | **通常一天内，您累计有多少时间进行上述高强度的锻炼或娱乐活动？** | | □□小时  □□分钟 |
| **E3.3** | | **您是否进行持续至少10 分钟，引起呼吸、心跳轻度增加的中等强度锻炼或娱乐活动吗？如快步走、打太极拳等。**  ⑴有 ⑵无**（跳至E4）** | |  |
| E3.4 | | **通常一周内，您有多少天进行上述中等强度的锻炼或娱乐活动？** | | □天 |
| E3.5 | | **通常一天内，您累计有多少时间进行上述中等强度的锻炼或娱乐活动？** | | □□小时  □□分钟 |
| **E4** | | **闲暇时，您每天坐着、靠着或躺着（如看电视、用电脑、阅读、写字、吃饭、打麻将、打牌、下棋等，不包括睡眠）的累计时间是多少？** | | □□小时  □□分钟 |
| E4.1 | | **您每天睡眠情况:**  ⑴睡眠感觉良好 ⑵睡眠感觉不好 ⑶安眠药助眠 | |  |
| E4.2 | | **您每天睡眠时间:** | | □□小时  □□分钟 |
| E4.3 | | **您午睡吗?**  ⑴是 ⑵否 | |  |
| **F. 饮食习惯** | | | |  |
| F1 | | **你家食用盐的种类？** ⑴碘盐 ⑵无碘盐 | |  |
| F1.1 | | **持续食用时间？** ⑴10年以上 ⑵7-9年 ⑶4-6年 ⑷1-3年 | |  |
| F1.2 | | **你家做菜的习惯是先放盐还是后放盐？**⑴先放 ⑵加工过程中放 ⑶后放 | |  |
| F1.3 | | **您的口味是:**  ⑴偏淡 ⑵一般 ⑶偏咸 | |  |
| F2  F3 | | **过去30天您食用下列食品的情况是？**  ⑴每天都吃 ⑵4-6天/周 ⑶1-3天/周 ⑷每月吃数次 ⑸不吃/极少吃  频率(选项见上) 每次量（两）  a)肉类及制品 □ □□  b)家禽及制品 □ □□  c)水产/海鲜 □ □□  d)海带/紫菜 □ □□  e)鲜蛋类 □ □□  f)新鲜蔬菜 □ □□  g)豆制品 □ □□  h)新鲜水果 □ □□  i)乳类制品 □ □□  j)腌制蔬菜 □ □□  k)玉米 □ □□  l)其他杂粮 □ □□ | **十年前您食用下列食品的情况是？**  ⑴每天都吃 ⑵4-6天/周 ⑶1-3天/周 ⑷每月吃数次 ⑸不吃/极少吃  频率(选项见上) 每次量（两）  a)肉类及制品 □ □□  b)家禽及制品 □ □□  c)水产/海鲜 □ □□  d)海带/紫菜 □ □□  e)鲜蛋类 □ □□  f)新鲜蔬菜 □ □□  g)豆制品 □ □□  h)新鲜水果 □ □□  i)乳类制品 □ □□  j)腌制蔬菜 □ □□  k)玉米 □ □□  l)其他杂粮 □ □□ |  |
| F4 | | **下列食品您经常吃吗？**⑴从不吃 ⑵很少吃 ⑶有时吃 ⑷经常吃 (请在下列内填入相应序号)  a)油炸食品（油条等） □  b)烟熏食品（熏肉、鱼等） □  c)腌晒食品（酸菜、泡菜等） □  d)炙烘烤食品（烤鸡、肉等） □  e)盐渍食品（咸菜、咸鱼肉等） □  f)腊味食品（腊肠等） □  g)辛辣味 □  h)酸味（醋类） □  i)大蒜类 □  j)葱类 □ | |  |
| F5 | | **您家做菜喜欢用什么方法？**⑴从不 ⑵很少 ⑶有时 ⑷经常 (请在下列内填入相应序号)  a)凉拌或生吃 □  b)炒 □  c)煎炸 □ d)蒸 □  e)熬煮 □  f)烤 □ | |  |
| **G. 环境暴露史** | | | |  |
| G1 | | **在您的长期住处或工作场所附近有无下列企业或设置？**  ⑴是**（填下表）** ⑵无**(跳至G2)** ⑼不详**(跳至G2)**  类别：⑴ 农药厂 ⑵ 化工厂 ⑶电镀厂 ⑷化肥厂 ⑸水泥厂 ⑹核电站 ⑺高压线或变压站 ⑻手机信号塔 ⑼其他   \| 类别 \| 距离（公里） \| 已存在年数 \| \| --- \| --- \| --- \| \| □ \| □□.□ \| □□ \| \| □ \| □□.□ \| □□ \| \| □ \| □□.□ \| □□ \| \| □ \| □□.□ \| □□ \| | |  |
| **G2** | | **职业接触:** ⑴是 ⑵否**(跳至G3)** | |  |
| G2.1 | | 1)电离辐射 2)甲醛 3)苯 4）金属粉末 5)锌 6) 硒 7）钒  最长时间 □□ 起始年份□□□□ 结束年份□□□□ 间断年数□□  第二长时间 □□ 起始年份□□□□ 结束年份□□□□ 间断年数□□  第三长时间 □□ 起始年份□□□□ 结束年份□□□□ 间断年数□□ | |  |
| G3 | | **是否使用手机？** ⑴是 ⑵否**(跳至G4)** | |  |
| G3.1 | | **使用多少年？** | | □□年 |
| G3.2 | | **每天手机通话_______分钟，上网_______分钟** | | □□□分钟  □□□分钟 |
| G3.3 | | **是否有下列手机使用习惯(可多选)**：  ⑴睡觉时开机放枕头边 ⑵平时放随身包里 ⑶平时放上衣口袋里  ⑷平时放裤子口袋里或腰带上 ⑸不用时尽量远离身体半米以上 | |  |
| **G4** | | **每天电脑使用时间：**  ⑴基本不用 ⑵半小时以下 ⑶一小时左右 ⑷数小时 ⑸说不清楚 | |  |
| G5 | | **平均每年接受X线检查或治疗** **次，持续** **年** | | □□次  □□年 |
| G5.1 | | **平均每年接受CT检查** **次，其中有颈部保护措施** **次，持续** **年** | | □□次  □□次  □□年 |
| G5.2 | | **检查原因是(可多选)**：⑴集体体检 ⑵个人预防性体检 ⑶因病检查 ⑷其他 | |  |
| G6 | | **您一生中坐飞机的次数：** | | □□次 |
| **H. 精神心理因素** | | | |  |
| H1 | | **性格：**⑴A型（动作快性急进取心强易激动） ⑵B型（与A相反） ⑶ C型 | |  |
| H2 | | **情绪：**⑴自我调节强,易适应环境 ⑵自我调节较差,不易适应环境 | |  |
| H3 | | **婚姻生活：**⑴和谐 ⑵一般 ⑶不和谐 ⑷破裂 | |  |
| H4 | | **人际关系：**⑴良好 ⑵一般 ⑶较差 | |  |
| H5 | | **精神状况：**你是否很长时间精神处于压抑状况？ ⑴是 ⑵否 | |  |
| H6 | | **近些年有无较大的精神创伤：** ⑴有 ⑵无 **(跳至I1)** | |  |
| H6.1 | | **如有，原因：** ⑴工作失意 ⑵亲人去世 ⑶家庭不和破裂 ⑷事故 ⑸其它 | |  |
| **I. 女性生理与生育史** | | | |  |
| **I1** | | **请问您月经初潮时的年龄？** | | □□周岁 |
| I1.1 | | **您月经经期持续天数** **天，月经间隔** **天** | | □□天  □□天 |
| I1.3 | | **您月经周期是否规律？** ⑴ 规律 ⑵不规律 | |  |
| I1.4 | | **您有痛经吗？** ⑴有 ⑵无 | |  |
| I2 | | **您怀过孕吗?**  ⑴有 ⑵无**（跳至I4）** | |  |
| I2.1 | | **怀孕结局及次数：**  ⑴自然流产□次 ⑵人工流产□次 ⑶活产□次 ⑷死产、死胎□次 ⑸畸形□次 ⑹其它□次 | |  |
| I2.2 | | **您第一次怀孕多大年龄?** | | □□周岁 |
| I2.3 | | **您第一次怀孕结果如何?** ⑴活产 ⑵死产 ⑶宫外孕 ⑷流产 ⑸人工流产 | |  |
| I2.4 | | **您末次怀孕多大年龄?** | | □□周岁 |
| I3 | | **您有无哺乳？** ⑴有 ⑵无**（跳至I4）** | |  |
| I3.1 | | **您哺乳孩子个数：** | | □个 |
| I3.2 | | **哺乳累计月数：** | | □□月 |
| I3.3 | | **您乳汁的情况？** ⑴多 ⑵一般 ⑶少 | |  |
| **I4** | | **您有无避孕？** ⑴有 ⑵无**（跳至I4.6）** | |  |
| I4.1 | | **开始避孕的年龄？** | | □□周岁 |
| I4.2 | | **避孕多长时间？** | | □□年 |
| I4.3 | | **主要避孕方法:** ⑴绝育 ⑵宫内节育器 ⑶口服药物 ⑷避孕膜、帽  ⑸皮下埋植 ⑹其它 | |  |
| I4.4 | | **如用药物避孕，共有多少年？** | | □□年 |
| I4.5 | | **药物种类：**⑴短效口服 ⑵长效口服 ⑶口服探亲  ⑷注射避孕针 ⑸事后避孕药 ⑹其它 | |  |
| **I4.6** | | **您有无服用过其他雌激素类药物?**  ⑴有 ⑵无**（跳至I5）** | |  |
| I4.7 | | **如果是，服用了下列哪种药物及使用年限?**(**可多选**)  1)维尼安(尼尔雌醇) □□年 2)倍美力 □□年  3)利维爱 □□年 4)诺坤复 □□年  5)补佳乐 □□年 6)苯甲酸雌二醇(针剂) □□年  7)己烯雌酚(针片剂) □□年 8)达英一 □□年  9)静心口服液(保健品)□□年 10)其他 □□年 | |  |
| **I5** | | **您是否接受过以下妇科相关手术？**  **是 否 如是，手术时年龄**  子宫切除术 □ □ □□周岁  卵巢摘除术 □ □ □□周岁  乳房肿块切除术 □ □ □□周岁  绝育术 □ □ □□周岁  破腹产 □ □ □□周岁 | |  |
| **J. 身体状况** | | | |  |
| J1 | | **身高：**□□□.□厘米 | |  |
| J2 | | **体重：**□□□.□公斤 | |  |
| J2.1 | | **生病前正常体重：**□□□.□公斤（**仅病例填写**） | |  |
| J3 | | **腰围****（厘米）：**□□□.□ | |  |
| J4 | | **臀围（厘米）：**□□□.□ | |  |
| J5 | | **血压（mmHg）：** 收缩压 □□□ 舒张压 □□□ | |  |
| **调查员后记** | | | |  |
|  | | **调查对象合作情况：** ⑴很好 ⑵好 ⑶一般 ⑷差 | |  |
|  | | **调查员对调查的评价？** ⑴可信 ⑵尚可信 ⑶不可信 | |  |

调查结束时间（24小时制）： □□时□□分

| **病例有关情况（查阅医院病历资料，对照组不需填写）：** | |  |
| --- | --- | --- |
| K0 | 发现方式是： ⑴体检 ⑵自检 ⑶其他 |  |
| K1 | 首次确诊日期：**（不详年月日均填满“9”）** □□□□年□□月□□日 |  |
| K2 | 诊断名称： |  |
| K2.1 | 确诊单位：⑴省级 ⑵地市级 ⑶县（区）级 ⑷乡级 ⑼不详 |  |
| K3 | 本次住院号： |  |
| K4 | 是否转移癌： ⑴是 ⑵否 ⑼不知道 |  |
| K5 | 是否多发？ ⑴是 ⑵否 ⑼不知道 |  |
| K6 | 肿瘤大小： / / （cm） **（若多发，填写最大肿瘤的大小）** | □□/□□/□□ |
| K7 | 组织学类型：  ⑴乳头状腺癌 ⑵滤泡状腺癌 ⑶髓样癌 ⑷未分化癌 ⑸其他 |  |
| K8 | 肿瘤细胞分化程度：  ⑴原位 ⑵低分化G1 ⑶中分化G2 ⑷高分化G3  ⑸其他 请注明： ⑼ 不详 |  |
| K9 | 诊断依据：**（可多选）**  ⑴病理 ⑵细胞学 ⑶免疫 ⑷生化 ⑸甲胎蛋白  ⑹手术 ⑺内窥镜 ⑻支气管镜 ⑼核磁共振 ⑽CT  ⑾临床 ⑿X线 ⒀超声波 ⒁其他 （99）不详 |  |
| K10 | TNM病理分期： T N M  ⑴0期 ⑵ⅠA期 ⑶ⅠB期 ⑷ⅡA期 ⑸ⅡB期  ⑹ⅢA期 ⑺ⅢB期 ⑻Ⅳ期 ⑼不详 |  |
| K11 | 是否转移： ⑴ 是 ⑵ 否**（跳至K13）** ⑼不详**（跳至K13）** |  |
| K12 | 转移部位： |  |
| K13 | 是否手术治疗： ⑴ 是 ⑵ 否 **（跳至K18）** |  |
| K14 | 手术名称： |  |
| K15 | 手术方式：  ⑴治性切除术 ⑵姑息性切除术 ⑶探查 ⑷其他 请注明 |  |
| K16 | 手术日期： □□□□年□□月□□日 |  |
| K17 | 手术医院：  手术医院级别： ⑴省级 ⑵地市级 ⑶县（区）级 ⑷乡级 ⑼不详 |  |
| K18 | 是否进行放射治疗（放疗）： ⑴ 是 ⑵ 否 **（跳至K21）** |  |
| K19 | 放射治疗时间：□□□□年□□月□□日/□□□□年□□月□□日**(可添加)** |  |
| K20 | 放射治疗单位： 放疗方案： |  |
| K21 | 是否进行化学药物治疗（化疗）： ⑴是 ⑵否（**跳至K24）** |  |
| K22 | 化疗时间： □□□□年□□月□□日/□□□□年□□月□□日**(可添加)** |  |
| K23 | 化疗单位： 化疗方案： |  |
| K24 | 是否进行中医药治疗？ ⑴ 是 ⑵ 否 ⑼不详 |  |
| K25 | 摘录临床检验结果：   \| 指标 \| 结果 \| \| --- \| --- \| \| 游离三碘甲状腺素(FT3) \|  \| \| 游离四碘甲状腺素(FT4) \|  \| \| 促甲状腺素(TSH) \|  \| \| 总甲状腺素(TT4) \|  \| \| 总三碘甲状腺原氨基酸(TT3) \|  \| \| 抗甲状腺氧化物酶自身抗体(TPOAb) \|  \| \| 抗促甲状腺素受体抗体(TRAb) \|  \| \| 甲状腺球蛋白(Tg) \|  \| \| ER \|  \| \| PR \|  \| \| HER-2 \|  \| \| 其他指标 \|  \| |  |
