# Supplementary material for: Menstrual, reproductive and hormonal factors and thyroid cancer: a hospital-based case–control study in China
Source: BMC Womens Health. 2021 Jan 6;21:13. doi: 10.1186/s12905-020-01160-w (PMC7789638; doi:10.1186/s12905-020-01160-w)
Supplement: Supplementary file 2 — Additional file 2: Table S1. ORs of thyroid cancer associated with menstrual, reproductive and hormonal factors, stratified by age at enrollment. [file 12905_2020_1160_MOESM2_ESM.docx]

| Additional file 1: Table S1. ORs of thyroid cancer associated with menstrual, reproductive and hormonal factors, stratified by age at enrollment. | | | | | | | | | | |
| --- | --- | --- | --- | --- | --- | --- | --- | --- | --- | --- |
| Factors | Overall | |  | Age ≦ 50 years | |  | Age > 50 years | |  | P value for interaction^∆^ |
|  | OR^†^ | 95%CI |  | OR^†^ | 95%CI |  | OR^†^ | 95%CI |  |  |
| *Menstrual factors* |  |  |  |  |  |  |  |  |  |  |
| Age at menarche (years) |  |  |  |  |  |  |  |  |  | 0.488 |
| ≦12 | 0.67 | 0.15-3.10 |  | 0.09 | 0.01-1.70 |  | 0.55 | 0.22-1.39 |  |  |
| 13-14 | Ref. |  |  | Ref. |  |  | Ref. |  |  |  |
| 15-16 | 0.79 | 0.48-1.30 |  | 0.69 | 0.20-2.42 |  | 1.04 | 0.76-1.44 |  |  |
| ≥17 | 0.59 | 0.31-1.12 |  | 0.20 | 0.02-2.38 |  | 0.89 | 0.61-1.30 |  |  |
| Had regular menstrual cycles |  |  |  |  |  |  |  |  |  | 0.083 |
| No | 0.61 | 0.28-1.35 |  | 0.36 | 0.06-2.02 |  | 0.82 | 0.47-1.43 |  |  |
| Yes | Ref. |  |  | Ref. |  |  | Ref. |  |  |  |
| Had dysmenorrhea |  |  |  |  |  |  |  |  |  | 0.986 |
| No | Ref. |  |  | Ref. |  |  | Ref. |  |  |  |
| Yes | 0.70 | 0.42-1.18 |  | 0.67 | 0.19-2.39 |  | 0.80 | 0.57-1.11 |  |  |
| Age at menopause (years) |  |  |  |  |  |  |  |  |  | 0.797 |
| ≦44 | 0.98 | 0.42-2.29 |  | 2.87 | 0.54-15.10 |  | 1.06 | 0.57-1.96 |  |  |
| 45-49 | 0.92 | 0.52-1.61 |  | 3.13 | 0.66-14.93 |  | 0.90 | 0.63-1.29 |  |  |
| 50-51 | Ref. |  |  | Ref. |  |  | Ref. |  |  |  |
| ≥52 | 0.89 | 0.55-1.44 |  | NA |  |  | 0.97 | 0.70-1.35 |  |  |
| *Reproductive factors* |  |  |  |  |  |  |  |  |  |  |
| Number of pregnancy |  |  |  |  |  |  |  |  |  | 0.829 |
| 1 | Ref. |  |  | Ref. |  |  | Ref. |  |  |  |
| 2 | 0.67 | 0.37-1.23 |  | 0.69 | 0.41-1.18 |  | 1.00 | 0.66-1.52 |  |  |
| ≥3 | 0.66 | 0.34-1.28 |  | 0.87 | 0.46-1.63 |  | 0.83 | 0.53-1.30 |  |  |
| Age at first pregnancy (years) |  |  |  |  |  |  |  |  |  | 0.391 |
| ≦20 | Ref. |  |  | Ref. |  |  | Ref. |  |  |  |
| 20-25 | 0.96 | 0.57-1.61 |  | 0.87 | 0.47-1.61 |  | 0.94 | 0.66-1.33 |  |  |
| >25 | **0.47** | **0.23-0.96** |  | 0.50 | 0.22-1.15 |  | 0.66 | 0.39-1.11 |  |  |
| Outcome of the first pregnancy |  |  |  |  |  |  |  |  |  | 0.749 |
| Live birth | Ref. |  |  | Ref. |  |  | Ref. |  |  |  |
| Miscarriage | 0.85 | 0.33-2.20 |  | 0.83 | 0.38-1.80 |  | 1.38 | 0.61-3.12 |  |  |
| Abortion | 0.80 | 0.30-2.09 |  | 1.07 | 0.50-2.27 |  | 1.62 | 0.69-3.84 |  |  |
| Stillbirth or ectopic | 1.37 | 0.33-5.62 |  | NA |  |  | 0.79 | 0.24-2.63 |  |  |
| Age at last pregnancy (years) |  |  |  |  |  |  |  |  |  | 0.245 |
| ≦25 | Ref. |  |  | Ref. |  |  | Ref. |  |  |  |
| 25-30 | 1.21 | 0.77-1.90 |  | 1.19 | 0.69-2.05 |  | 0.85 | 0.61-1.18 |  |  |
| >30 | 1.63 | 0.88-3.00 |  | 0.91 | 0.48-1.72 |  | 0.93 | 0.61-1.43 |  |  |
| Duration of breast feeding (months) |  |  |  |  |  |  |  |  |  | 0.486 |
| ≦6 | Ref. |  |  | Ref. |  |  | Ref. |  |  |  |
| 6-12 | **0.49** | **0.24-0.98** |  | **0.51** | **0.29-0.89** |  | 0.59 | 0.33-1.07 |  |  |
| >12 | 0.60 | 0.27-1.31 |  | 0.73 | 0.36-1.47 |  | 0.55 | 0.30-1.02 |  |  |
| *Hormonal factors* |  |  |  |  |  |  |  |  |  |  |
| Oral contraceptive use |  |  |  |  |  |  |  |  |  | 0.288 |
| No | Ref. |  |  | Ref. |  |  | Ref. |  |  |  |
| Yes | 1.18 | 0.47-2.96 |  | 1.07 | 0.38-3.03 |  | 1.09 | 0.52-2.29 |  |  |
| Hormone therapy use |  |  |  |  |  |  |  |  |  | 0.388 |
| No | Ref. |  |  | Ref. |  |  | Ref. |  |  |  |
| Yes | 1.47 | 0.30-7.38 |  | 1.88 | 0.54-6.62 |  | 1.25 | 0.45-3.47 |  |  |
| Hysterectomy and oophorectomy status |  |  |  |  |  |  |  |  |  | 0.149 |
| None | Ref. |  |  | Ref. |  |  | Ref. |  |  |  |
| Hysterectomy alone | 1.45 | 0.77-2.73 |  | 0.49 | 0.19-1.27 |  | **1.90** | **1.13-3.19** |  |  |
| Oophorectomy alone | 1.93 | 0.43-8.72 |  | 0.40 | 0.09-1.77 |  | 2.96 | 0.62-14.11 |  |  |
| Hysterectomy and oophorectomy | 3.85 | 0.43-34.59 |  | NA |  |  | 1.12 | 0.26-4.91 |  |  |
| OR odds ratio, CI confidence interval, Ref. reference, NA No available due to the limited number of subjects. Bold numbers represent significant results.  † Model 4: adjusted for education level, average monthly household income, marriage status, history of goiter and nodules, alcohol intake and body mass index.  ^∆^ Effect modification was tested by adding interaction terms between these study factors and age independently to the model. | | | | | | | | | | |
